# Development and validation of a prognostic nomogram model for 28-day mortality based on IP-10 and clinical parameters in septic patients in the emergency department

**DOI:** 10.3389/fcimb.2026.1838120

**Published:** 2026-09-07

**Authors:** Long Yang, Yue Lin, Xiangqun Zhang, Guijuan Dong, Na Shang, Wenpeng Yin, Junyu Wang, Xinhua He, Xue Mei

**Affiliations:** 1 Beijing Chao-Yang Hospital Emergency Medical Center Affiliated to Capital Medical University, Beijing Key Laboratory of Cardiopulmonary-Cerebral Resuscitation Innovation and Translation, Beijing, China; 2 Department of Radiology, China-Japan Friendship Hospital, Beijing, China

**Keywords:** 28-day mortality, APACHE II score, IP-10, LASSO, nomogram

## Abstract

**Background:**

Early identification and precise prognosis of sepsis are of great significance. Traditional biomarkers, such as procalcitonin (PCT) and lactate (Lac), do not reflect the immune dysregulation at the center of sepsis pathophysiology. Interferon - γ -induced protein 10 (IP-10) is an important chemokine for septic immune dysregulation responses and may have good predictive value. Our research aims to establish and validate a clinically applicable nomogram that combines IP-10 and conventional clinical parameters to predict the 28-day mortality in septic patients in the emergency department (ED).

**Methods:**

This study recruited 655 patients who met the criteria of sepsis 3.0 from the ED of Beijing Chao-Yang Hospital from November 2023 to May 2025. A total of 580 patients were analyzed and randomly divided into the training group and the validation group in a 7:3 ratio. The variable selection in this study was conducted using the Least Absolute Shrinkage and Selection Operator (LASSO) regression, and our research employed multivariate logistic regression to determine independent predictors of 28-day mortality. The study scientifically evaluated nomogram using receiver operating characteristic (ROC) curves, calibration plots, Decision Curve Analysis (DCA), and Clinical Impact Curves (CIC).

**Results:**

Among the 580 patients, the 28-day mortality rate was 12.9% (75 non-survivors). Multivariate analysis identified five independent risk factors: IP-10 (OR = 1.978, 95% CI: 1.964-1.992), Sequential Organ Failure Assessment (SOFA) score (OR = 1.526, 95% CI: 1.365-1.758), Lac (OR = 1.447, 95% CI: 1.281-1.711), PCT (OR = 1.562, 95% CI: 1.341-1.924), and Acute Physiology and Chronic Health Evaluation II (APACHE II) score (OR = 1.658, 95% CI: 1.515-1.840). The nomogram showed excellent discriminatory ability. The area under the curve (AUC) of the training set and validation set was 0.952 (95% CI: 0.930-0.974) and 0.946 (95% CI: 0.911-0.981), respectively. Calibration was satisfactory, and DCA/CIC analyses confirmed that within the risk threshold range, the clinical net benefit was significant.

**Conclusion:**

It is the first time to develop and validate a prognostic nomogram model for 28-day mortality based on IP-10 and clinical parameters in septic patients in the ED. This tool may assist clinicians in early risk stratification and clinical decision-making.

## 1 Introduction

Sepsis is characterized by life-threatening organ dysfunction due to the host’s maladaptive response to infection, which imposes a significant burden on global health (Girard and Ely, 2007), and is implicated in nearly 20% of all deaths worldwide. Patients with sepsis are often in critical condition and their illness progresses rapidly, leaving doctors with little time to react. Early identification and reasonable treatment can significantly reduce the mortality (Kethireddy et al., 2018).

Traditionally, the current prognostic tools in the emergency department largely rely on common inflammatory indicators and clinical scoring systems. The most common and widely used one is the SOFA score. The SOFA score is a component of the Sepsis-3 definition (Singer et al., 2016), which can quantitatively assess organ dysfunction, but it cannot fully reflect early physiological disorders. Although qSOFA (quick sepsis-related organ failure assessment) (Raith et al., 2017) is simple, its sensitivity changes. It is mainly used as a clinical rapid screening tool and not as the main evaluation index for clinical prognosis. Lactate indicates insufficient overall tissue perfusion and microcirculation disorders, but it has a relatively low response to the immune pathological characteristics of sepsis. This dysregulated host response involves an initial hyper inflammatory phase often followed by protracted immunosuppression, a key driver of late mortality. Consequently, a biomarker reflective of this immune dysregulation could enhance prognostic accuracy.

Interferon-gamma-induced protein 10 (IP-10/CXCL10) is a chemokine induced by interferon-gamma and other pro-inflammatory signals (Álvarez et al., 2023). IP-10 is usually secreted by a variety of different cells, including endothelial cells, monocytes and fibroblasts, etc. IP-10 is expressed in many TH1-type inflammatory diseases. Among these diseases, IP-10 plays a significant role in recruiting and activating T cells to enter the inflammatory sites of tissues (Dufour et al., 2002; Foley et al., 2005; Ploquin et al., 2016; Wang et al., 2021). In sepsis, IP-10 remaining at a high level is associated with both the initial cytokine storm and the subsequent immunosuppressive phase, correlating with T-cell exhaustion and impaired monocyte function. Elevated IP-10 has been linked to increased mortality in sepsis (Punyadeera et al., 2010) and also observed in COVID-19 and HIV-1 infection (Simmons et al., 2013; Huang et al., 2020; Liu et al., 2020). However, its integration into a pragmatic bedside prognostic model for ED septic patients warrants further investigation.

In recent years, the advancement of statistical methods and computing techniques has promoted the rapid development of clinical prediction models. More and more machine learning algorithms have demonstrated higher predictive performance in the early risk assessment and stratification of sepsis (Zhang et al., 2021; Zhang et al., 2022a; Zhang et al., 2022b). Interpretable predictive models based on machine learning that can be visualized with nomograms are playing a significant role and are currently widely applied (Wang et al., 2025). Nomograms are graphical calculation tools that combine multiple predictors to generate individualized risk estimates, offering user-friendly clinical application (Jalali et al., 2019; He et al., 2022; Hu et al., 2025a; Jin et al., 2025). While some nomograms exist for sepsis prognosis, few are specifically designed for the ED setting where early risk assessment is most critical.

Therefore, the aim of this study is to develop and validate a new prognosis nomogram, incorporating IP-10 and key clinical parameters, to predict 28-day mortality specifically for septic patients upon ED admission.

## 2 Methods

### 2.1 Study population and design

This study was conducted in the ED of Beijing Chao-Yang Hospital. From November 2023 to May 2025, 655 patients (≥18 years) meeting Sepsis-3.0 criteria (suspected infection + SOFA score ≥2) were screened. Exclusion criteria comprised: age < 18 years, presence of end-stage diseases (metastatic malignancy, AIDS, end-stage renal or liver disease), refusal to participate, or incomplete clinical data. Total, 28 people refused to participate, and 47 people had missing or incomplete data. The proportion of missing data among the total number of people is 7.2% (47/655).

### 2.2 Data collection

Blood samples were collected at ED admission. Laboratory test indicators, including WBC (white blood cells), biochemistry, arterial blood gas, PCT, C-reactive protein (CRP), Lac, albumin, liver enzymes, and serum creatinine were measured within 24 hours. Serum IP-10 concentrations were determined using enzyme-linked immunosorbent assay (ELISA) kits. The specific IP-10 detection parameters were as follows. 1. Source of kit: R &D Systems, Catalog #: QK266. 2. Sensitivity: 4.1pg/ml. 3. Variance coefficient: Intra-assay: <10%; Inter-assay: <12%. 4. Sample retention conditions: Refrigerate at 2–8 degrees Celsius. All samples were collected in duplicate for storage purposes and for use in additional experiments if needed, to reduce errors and improve accuracy. SOFA and APACHE II scores were calculated based on clinical and laboratory data at presentation. All patients were followed for 28 days, with death from any cause as the primary endpoint.

### 2.3 Statistical analysis and model development

Statistical analysis was conducted using R software (version 4.3.2). For continuous variables, they were often expressed as mean ± standard deviation, median, or interquartile range. When making comparisons, Student’s t-test or Mann-Whitney U test were used. For categorical variables expressed in terms of frequency, chi-square test was used for comparison.

We used LASSO regression to select the most relevant predictors in the training cohort, minimizing overfitting. The variables of LASSO were brought into the multivariate logistic regression model to determine the independent risk factors and the ORs (odds ratios) were calculated with 95% CIs (confidence intervals).

This study established a nomogram based on the final multivariate model. The ROC curve and AUC value were used to evaluate its discriminative ability. The performance improvement of the nomogram compared to the SOFA score and APACHE II score was assessed using the Integrated Discrimination Improvement (IDI) and the Net Reclassification Improvement (NRI). Evaluate the calibration with the calibration diagram and the Hosmer-Lemeshow fit test. Clinical utility was examined through DCA and CIC. Internal validation was performed using bootstrap resampling (1000 iterations). Sample size calculations, 10-fold cross-validation and sensitivity analysis were performed to directly address overfitting concerns on the development cohort. Bootstrap resamples were performed to estimate the optimism-corrected performance of the model. A two-tailed *p*-value < 0.05 was considered statistically significant.

## 3 Results

### 3.1 Patient characteristics

**Figure 1:**
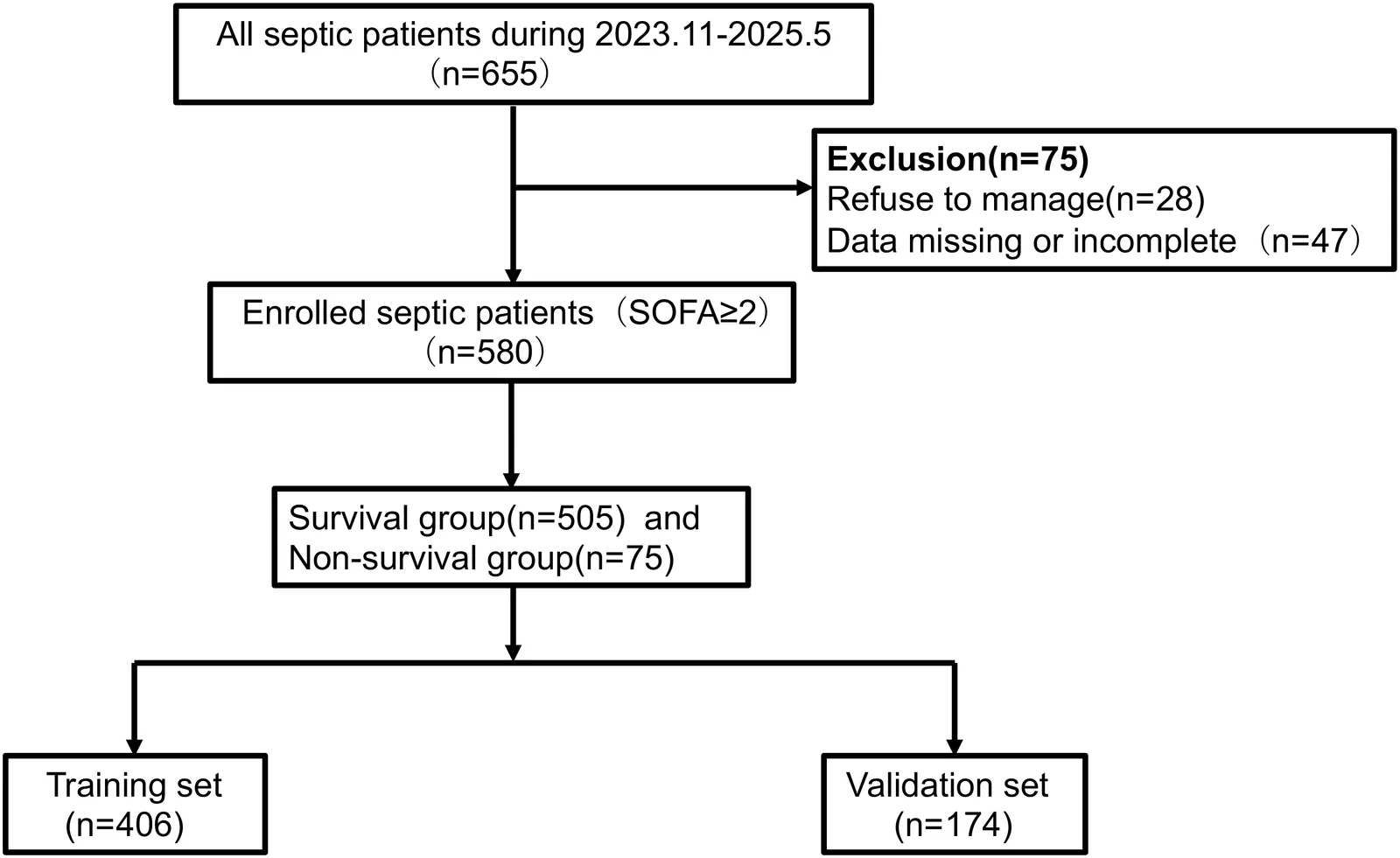
Flowchart of the study.

After exclusions (refuse to manage, n=28 and Data missing or incomplete, n=47), 580 patients were included in the final analysis (Training Set: n=406; Validation Set: n=174). The overall 28-day mortality rate was 12.9% (75/580) (**Figure 1**).

Notably, serum IP-10 levels at presentation were markedly elevated in non-survivors [median 119.9pg/mL (IQR: 59.1-189.9)] versus survivors [49.4pg/mL (IQR: 36.9-90.1)] (*p* < 0.001). There was no statistically significant difference in gender, comorbidities (cerebrovascular disease, hypertension, diabetes) (**Table 1**).

**Table 1:** Basic information of the study cohort.

Variables	Survivor (n=505)	Non-survivors (n=75)	P
Age, years	67 (62, 84)	75 (65, 83)	0.731
Male, %	597 (58.2)	90 (58.1)	0.966
COPD, %	164 (16.0)	36 (23.2)	0.025
Hypertension, %	420 (41.0)	61 (47.7)	0.702
CVD, %	164 (16.0)	30 (19.4)	0.294
CHF, %	373 (36.4)	88 (56.8)	0.000
DM, %	280 (27.3)	47 (30.3)	0.436
MAP, mmHg	101 (96, 104)	99 (89, 107)	0.341
Temperature, °C	36.3 (36, 36.7)	36.5 (36, 36.7)	0.877
HR, beats/min	86 (69, 98)	94 (79, 113)	0.269
RR, bpm	20 (17, 22)	24 (20, 28)	0.030
WBC, *10^9/L	8.3 (7.2, 9.8)	9.5 (7.7, 11.6)	0.138
PLT, *10^9/L	180 (144, 232)	169 (139, 246)	0.964
Lac, mmol/L	1.4 (0.9, 2.1)	2.9 (1.3, 5.8)	0.017
Scr, mmol/L	76 (59, 98)	68 (56, 99)	0.672
ALB, g/L	39 (35, 43)	31 (24, 38)	0.008
AST, U/L	25 (18, 37)	42 (24, 86)	0.030
ALT, U/L	18 (14, 25)	49 (26, 76)	0.001
PCT (ng/ml)	0.05 (0.05, 0.08)	1.50 (0.05, 2.78)	0.002
CRP (mg/L)	21 (8, 35)	55 (14, 97)	0.024
IP-10 (pg/ml)	49.4 (36.9, 90.1)	119.9 (59.1, 189.9)	0.000
SOFA	3 (2, 6)	8 (6, 10)	0.000
APACHE II	12 (9, 15)	24 (17, 33)	0.000

IP-10, Interferon gamma-induced protein 10; WBC, white blood cell; PCT, procalcitonin; Lac, lactate; CRP, C-reactive protein; ALB, albumin; Scr, serum creatinine; AST, aspartate aminotransferase; ALT, alanine transaminase; PLT, platelet; MAP, mean arterial pressure; RR, respiratory rate; HR, heart rate; SOFA, Sequential Organ Failure Assessment; APACHE II, Acute Physiology and Chronic Health Evaluation II; COPD, chronic obstructive pulmonary disease; CVD, cerebrovascular disease; CHF, congestive heart failure; DM, diabetes mellitus.

### 3.2 Predictor selection and model development

**Figure 2:**
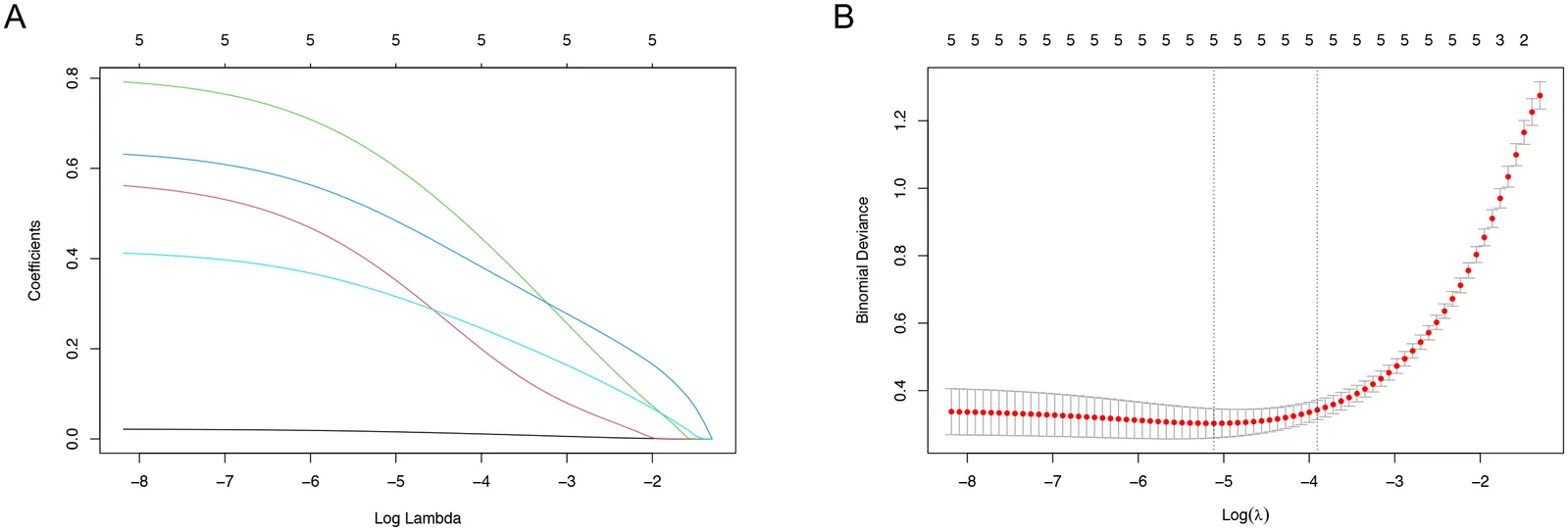
LASSO regression was used for demographic and clinical feature selection. **(A)** LASSO regression coefficient log(Lambda) profile; **(B)** LASSO regression cross-validation results.

LASSO regression analysis selected five non-zero coefficient variables: IP-10, SOFA score, Lac, PCT, and APACHE II score (**Figure 2**). Subsequent multivariate logistic regression confirmed that all five factors were independent predictors for 28-day mortality. (**Table 2** for ORs and CIs).

**Table 2:** Multivariate Logistic regression analysis of 28-day mortality for septic patients.

Variables	*β*	*SE*	Wald	*P*	OR(95%CI)
IP-10	-0.022	0.007	9.010	0.003	1.978 (1.964-1.992)
Lac	-0.805	0.237	11.526	0.001	1.447 (1.281-1.711)
PCT	-0.577	0.254	5.156	0.023	1.562 (1.341-1.924)
SOFA	-0.643	0.187	11.868	0.001	1.526(1.365-1.758)
APACHE II	-0.419	0.125	11.272	0.001	1.658 (1.515-1.840)
Constant	11.224	1.998	31.544	0.000	

IP-10, Interferon gamma-induced protein10; PCT, procalcitonin; Lac, lactate; SOFA, Sequential Organ Failure Assessment; APACHE II, Acute Physiology and Chronic Health Evaluation II.

### 3.3 Construction and presentation of the nomogram model

**Figure 3:**
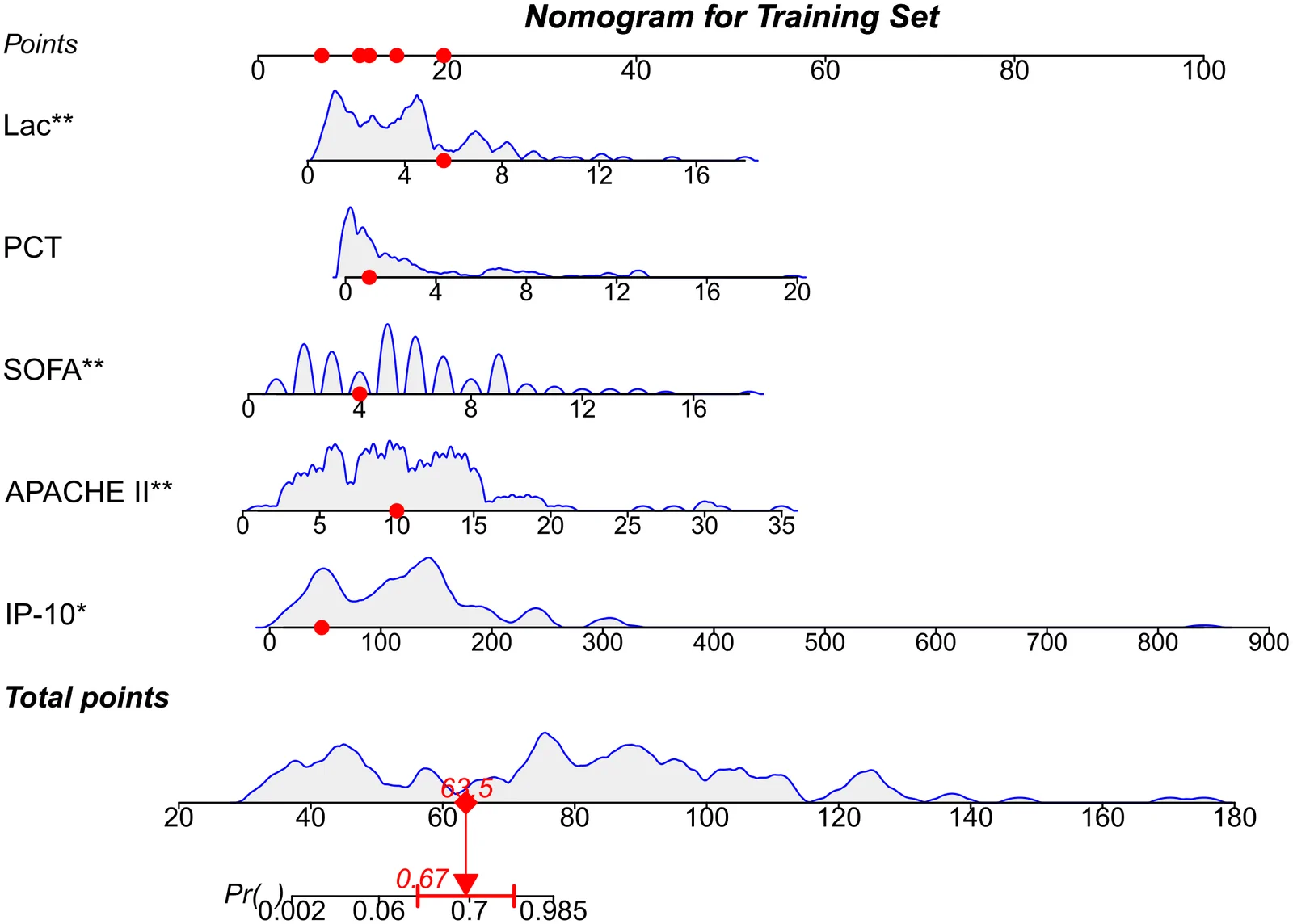
Nomogram model for 28-day mortality prediction of septic patients. IP-10(per 20pg/ml), SOFA score (per 1point), APACHE II score (per 1point), Lactate (per 1mmol/L), PCT (per 1ng/ml). *P < 0.05, **P < 0.01.

This study established a visual nomogram based on the final logistic regression model (**Figure 3**). Each predictor was assigned a score on a point scale. The scores of the five variables were added together to obtain a total score, which exactly corresponded to the prediction of the 28-day mortality on the bottom axis. The reading guideline and model application cases could be found in the supplementary materials (**Supplementary 1**).

### 3.4 Evaluation of the model

**Figure 4:**
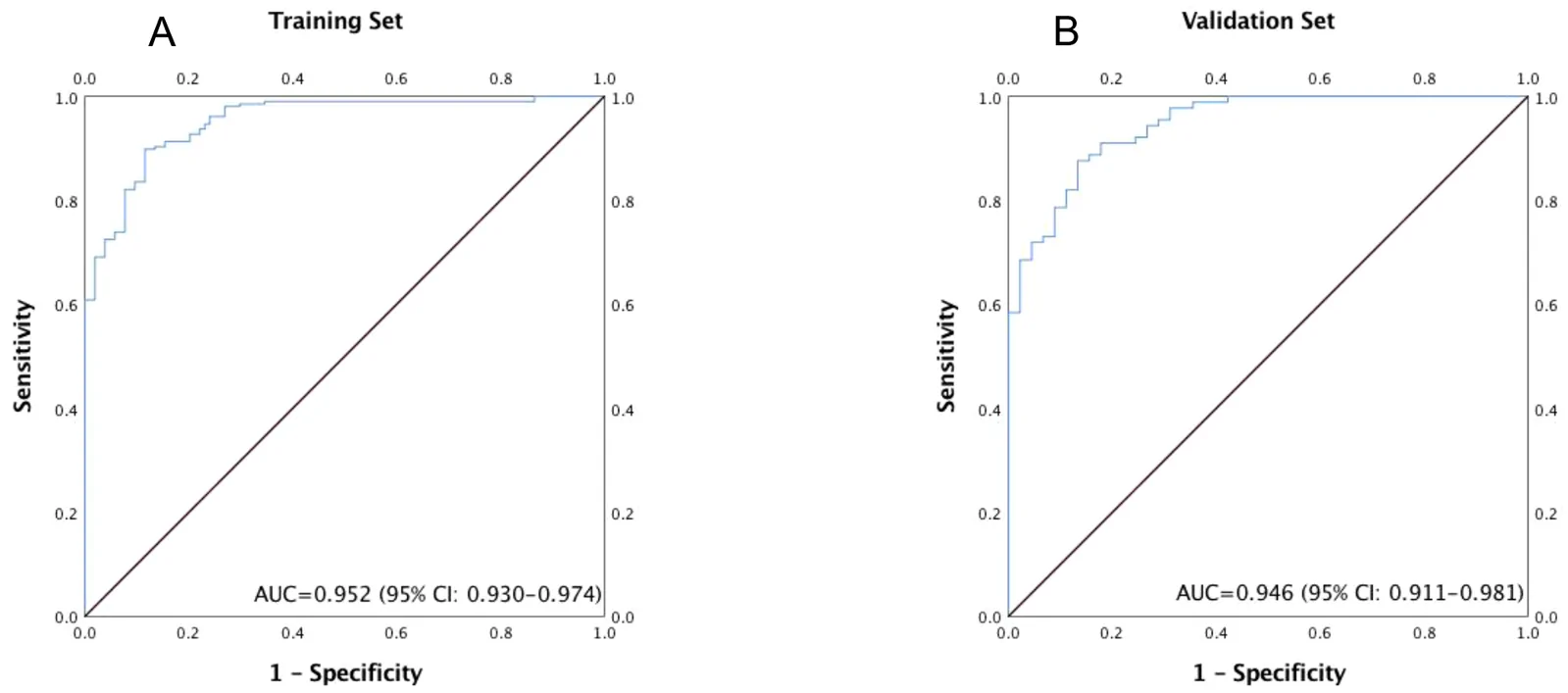
ROC curves for the risk prediction model in septic patients in the training set **(A)** and validation set **(B)**.

**Discrimination:** The discriminative ability of this nomogram was relatively good. The AUC of the training set and validation set was 0.952 (95% CI: 0.930-0.974) and 0.946 (95% CI: 0.911-0.981), respectively (**Figure 4**). These values were significantly higher than those for IP-10 (AUC: 0.880, *p* < 0.001), the SOFA score (AUC: 0.882, *p* < 0.001) or APACHE II score alone (AUC: 0.873, *p* < 0.001) (**Table 3**).

**Table 3:** Statistical data of ROC curve in predicting 28-day-mortality in septic patients.

Variables	AUC (95%CI)	*SE*	*P*	Cut off	Sensitivity	Specificity
IP-10	0.880(0.844-0.915)	0.018	0.000	95.5	0.993	0.985
SOFA	0.882 (0.849-0.915)	0.017	0.000	3.5	0.993	0.986
APACHE II	0.873 (0.838-0.909)	0.018	0.000	6.5	0.981	0.985
Lac	0.899 (0.866-0.931)	0.016	0.000	2.6	0.973	0.986
PCT	0.884(0.851-0.916)	0.017	0.000	0.5	0.959	0.986
Training set	0.952(0.930-0.974)	0.011	0.000		0.990	0.981
Validation set	0.946(0.911-0.981)	0.018	0.000		0.989	0.978

IP-10, Interferon gamma-induced protein10; PCT, procalcitonin; Lac, lactate; SOFA, Sequential Organ Failure Assessment; APACHE II, Acute Physiology and Chronic Health Evaluation II;.

#### 3.4.1 Calibration

**Figure 5:**
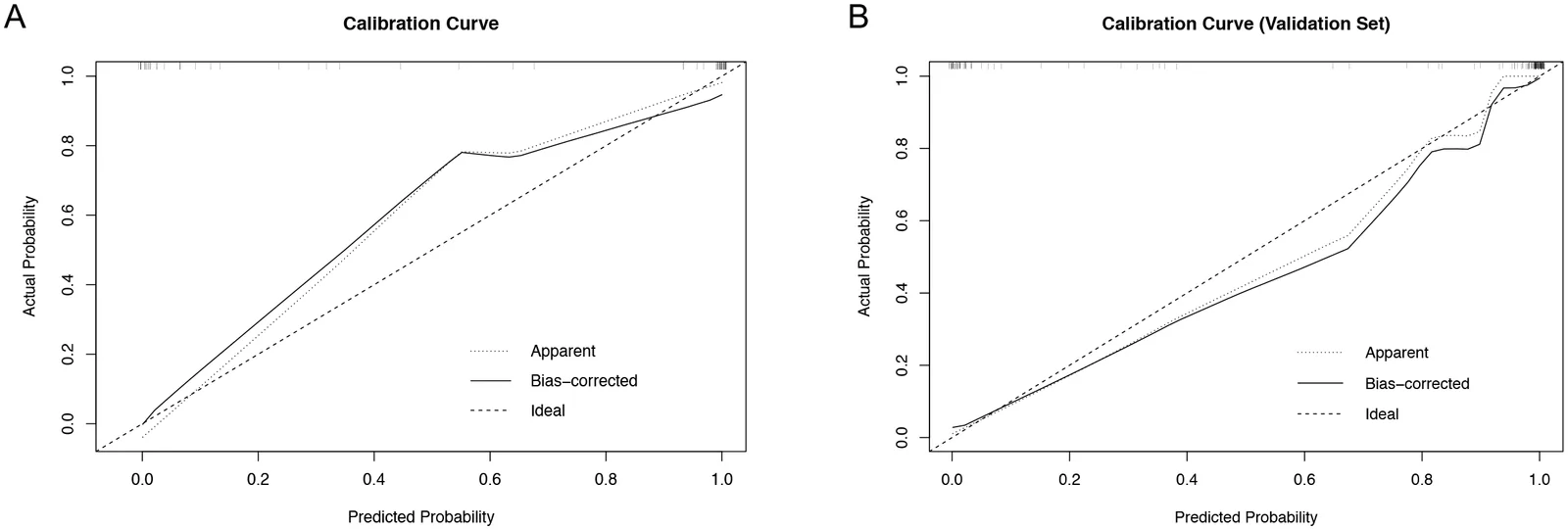
Calibration curves for the risk prediction model in septic patients in the training set **(A)** and validation set **(B)**.

The calibration curve showed that the predicted probabilities of the training set and the validation set had a good consistency with the observed results. The *P*-value of the Hosmer-Lemeshow test was not significant (*p*>0.05), indicating that our model fitted satisfactorily (**Figure 5**).

The nomogram showed significant improvements in risk reclassification compared with conventional scores. In the training cohort, categorical NRI values were 0.893 (95% CI, 0.570-1.103) versus SOFA score and 0.831 (0.550-1.067) versus APACHE II score (all *P* < 0.001). Continuous NRI values showed similar improvements: 1.778 (1.512-2.000) versus SOFA score and 1.766 (1.470-2.000) versus APACHE II score (all *P* < 0.001). IDI values also showed significant improvement: 0.446 (0.314-0.637) versus SOFA score and 0.499 (0.371-0.688) versus APACHE II score (all *P* < 0.001) (**Table 4**).

**Table 4:** Comparison of the performance of nomogram model in predicting 28-day-mortality in septic patients.

Predict model		AUROC	*P* value	NRI(categorical)	*P* value	NRI(Continuous)	*P* value	IDI	*P* value
Training set	Nomogram	0.952							
	SOFA	0.882	<0.001	0.893(0.570-1.103)	<0.001	1.778(1.512-2.000)	<0.001	0.446 (0.314-0.637)	<0.001
	APACHE II	0.873	<0.001	0.831(0.550-1.067)	<0.001	1.766(1.470-2.000)	<0.001	0.499(0.371-0.688)	<0.001
Validation	Nomogram	0.946							
set	SOFA	0.881	0.007	0.883(0.709-1.079)	<0.001	1.498(1.166-1.774)	<0.001	0.378 (0.272-0.465)	<0.001
	APACHE II	0.865	0.014	0.658(0.394-0.879)	<0.001	1.703(1.408-1.937)	<0.001	0.430(0.291-0.542)	<0.001

SOFA, Sequential Organ Failure Assessment. APACHE II, Acute Physiology and Chronic Health Evaluation II. AUROC, Area Under the ROC Curve. NRI, Net Reclassification Improvement. IDI, Integrated Discrimination Improvement.

To directly address overfitting concerns, we have conducted sample size calculations. Based on an estimated mortality rate of 20% and an anticipated AUC improvement of 0.05, a minimum of 150 death events were required to achieve precise validation. We should further increase the sample size and conduct external and prospective validation studies to reduce the risk of overfitting. Meanwhile, 10-fold cross-validation was performed on the development cohort. The cross-validated area under the curve (AUC) was 0.926, compared to the apparent AUC of 0.952 in the full-sample analysis. The degree of optimism was approximately 0.026, indicating mild but present overfitting. The generalization ability of the model needs further external verification.

As an additional sensitivity analysis, we performed 500 bootstrap resamples to estimate the optimism-corrected performance of the model. The apparent AUC was 0.952, and the optimism-corrected AUC was 0.926 (95% CI: 0.893–0.955). The optimism-corrected calibration slope was 0.94, suggesting acceptable calibration.

#### 3.4.2 Clinical utility

**Figure 6:**
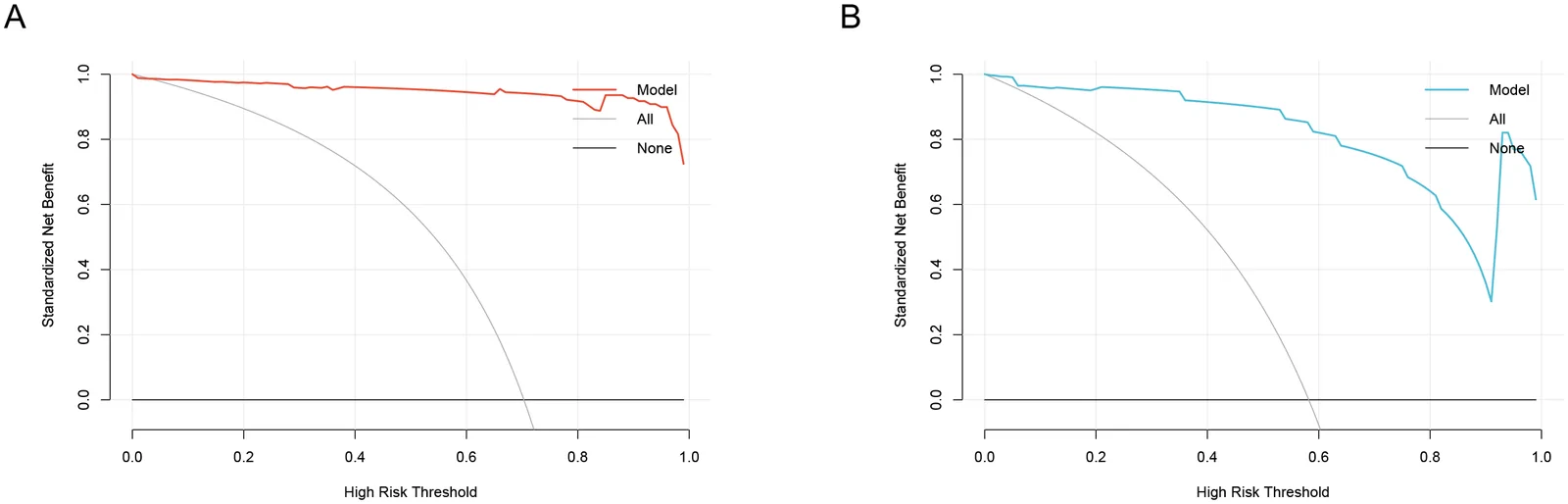
Decision curve analysis(DCA) for the risk prediction model in septic patients in the training set **(A)** and validation set **(B)**.

**Figure 7:**
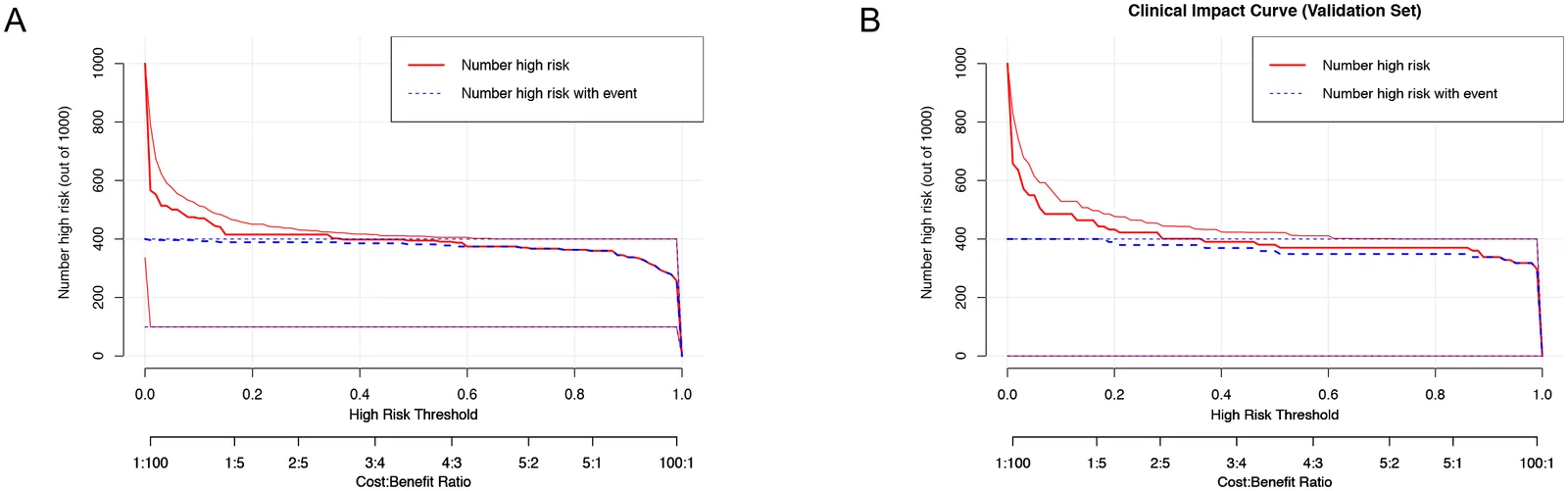
Cumulative incidence curves(CIC) for the risk prediction model in septic patients in the training set **(A)** and validation set **(B)**.

**Figure 8:**
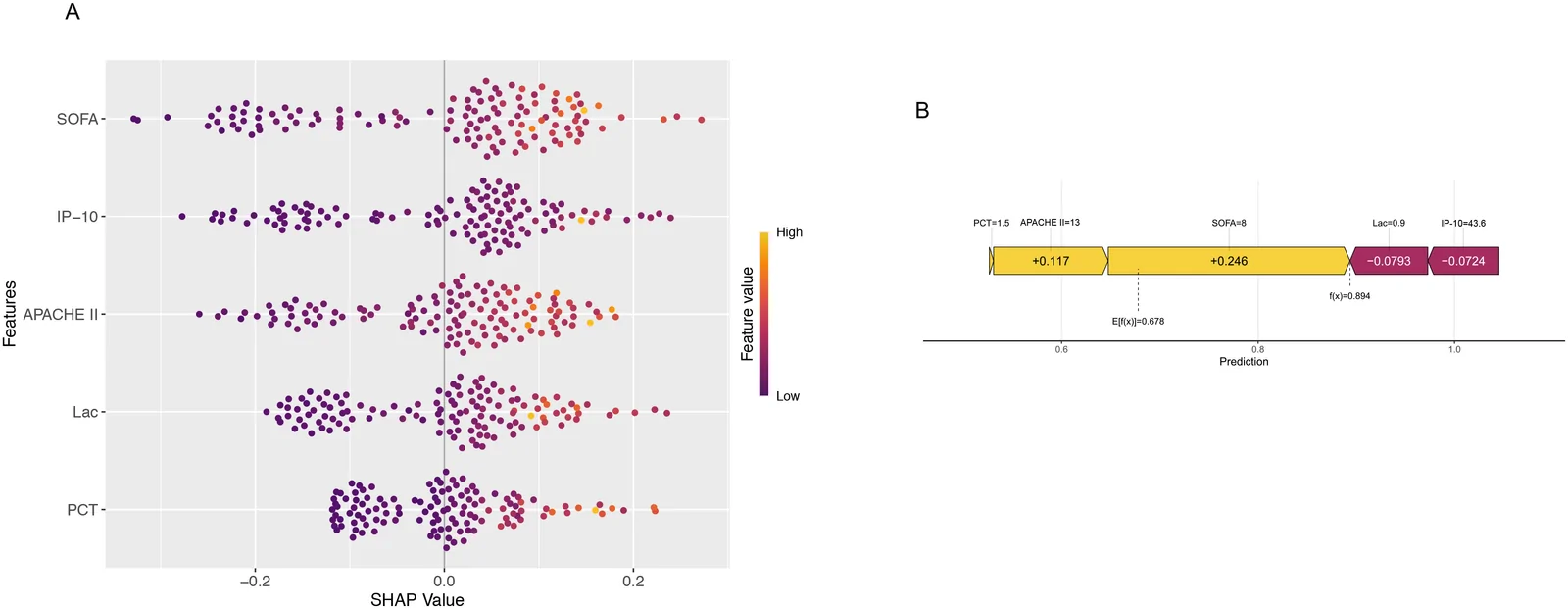
SHAP analysis for predicting 28-day mortality in septic patients. The beeswarm plot **(A)** shows the distribution of SHAP values for each feature, with color intensity indicating feature values. The force plot **(B)** illustrates the contribution of individual features to a specific prediction, showing how each feature affects the model’s output.

DCA showed that within the threshold probability range, the nomogram model offered higher net benefit for clinical decision-making compared to strategies that treat all or no patients (**Figure 6**). The results of the CIC showed that the number of deaths of high-risk patients predicted by the model was highly matched with the number of true positive deaths of high-risk patients (**Figure 7**). SHAP analysis identified SOFA score, IP-10 and APACHE II score as the strongest predictors of mortality (**Figure 8**).

## 4 Discussion

Our research established and validated a new prognostic nomogram integrating the immune biomarker IP-10 with established clinical parameters for 28-day mortality prediction in patients with sepsis in the ED. The model, comprising PCT, Lac, IP-10, SOFA score, and APACHE II score, demonstrated excellent discrimination, calibration, and clinical net benefit, outperforming commonly used prognostic scores.

Our findings strongly confirmed the independent prognostic value of IP-10 in sepsis. The median IP-10 level in non-survivors was more than double that in survivors, and it emerged as a powerful independent predictor. This aligned with previous smaller studies linking elevated IP-10 to mortality in sepsis (Punyadeera et al., 2010; Eichhorn et al., 2023). We know that IP-10 is an immune chemokine that plays a key role in recruiting and activating immune cells, such as T lymphocytes, to the site of inflammation (Vacharathit et al., 2025). It binds to the CXCR3 receptor, which is expressed on activated Th1 cells as well as some circulating CD4+ and CD8+ T cells (Loetscher et al., 1996; Sallusto et al., 1998; Xie et al., 1999). Its abnormal increase is associated with the pathophysiological changes of different chronic inflammations and autoimmune diseases (Vacharathit et al., 2025). Unlike indicators such as PCT and lactate that reflect infection burden or physiological disorder results, IP-10 directly points to the essence of sepsis, dysregulation of the host immune response. High levels of IP-10 are not only associated with initial excessive inflammation (cytokine storm), but also strongly related to subsequent immune paralysis states such as T cell exhaustion and monocyte dysfunction (Kaplan et al., 1987; Gupta et al., 2011), the latter of which is the key to late infection loss of control and death. As a kind of chemokine upstream in the immune pathway, its changes may occur earlier than the significant deterioration of organ function downstream, providing a theoretical time window for ultra-early intervention (such as immunomodulatory therapy). Therefore, a high IP-10 level at presentation may identify patients already on a trajectory of profound and potentially fatal immune paralysis, beyond what is captured by organ dysfunction scores alone.

The SOFA score is an important component of sepsis 3.0. Clinically, it is widely used to evaluate the severity of organ dysfunction and is a particularly important predictor in our model (Raith et al., 2017; Bauer et al., 2020). Designed for daily or sequential assessment, it could dynamically track treatment effects and provide real-time feedback for adjustments to treatment plans. The SOFA score has its limitations. Its dependence on the baseline status makes it difficult to accurately determine its acute changes, which may lead to missed diagnosis or delayed diagnosis. It is impossible to reveal the etiology and pathological mechanism, etc.

Elevated lactate levels reflect tissue hypo perfusion and cellular shock, key pathological processes in sepsis. Hyperlactatemia directly reflects hypo perfusion of systemic tissues and aerobic metabolic disorders at the cellular level (Revelly et al., 2005; Vincent et al., 2016). Lactate levels could be used as dynamic monitoring indicators for fluid resuscitation and vasoactive drug therapy. Lactate also has its limitations in predicting sepsis. It lacks disease specificity, can’t reveal the immune status, and is affected by multiple confounding factors, etc.

PCT serves as a marker of the systemic bacterial infection burden (Ruan et al., 2018). High specificity, highly correlated with bacterial infections. Compared with non-specific inflammatory indicators such as CRP, PCT significantly increases under the stimulation of bacterial infection (Godínez-Vidal et al., 2020; Pu et al., 2025), while the increase is limited in non-infectious inflammations (such as trauma, surgery, and the acute phase of autoimmune diseases). PCT has its predictive limitations. It has limited predictive value for non-bacterial infections and local infections. In sepsis caused by typical viral or invasive fungal infections, PCT may not increase or only increase slightly, at which point its predictive value decreases.

The APACHE II score provides a comprehensive assessment of acute physiological derangement. It integrates three major dimensions: acute physiological disorders, age and chronic health status, covering all organ systems (Tekin et al., 2024). It assesses the disruption of physiological homeostasis more extensively than SOFA score and is the most comprehensive critical illness scoring system. APACHE II score is the most widely used and recognized scoring system in ICU for population risk adjustment, comparison of medical quality and clinical research (Luo et al., 2021). The APACHE II score has its limitations in prediction. It is highly complex and has relatively poor timeliness. The calculation process is complex and it is often impossible to obtain them completely in the very early stage of the emergency department such as within one hour of admission.

Herein, we integrated the above parameters to create a multidimensional risk assessment tool. The SOFA score was incorporated into the model as an authoritative quantitative indicator of organ dysfunction, which ensured the validity and credibility of the model in assessing sepsis (Schertz et al., 2023). The powerful prediction was one of the cornerstones of the model’s performance. PCT was included as an authoritative biomarker of systemic bacterial infection burden and inflammatory response intensity. It provided important information on etiological specificity (bacterial infection) and disease severity, and was an indispensable part of the model. The APACHE II score was included as a comprehensive metric for evaluating the severity of overall acute physiological disorders, age and basic health status of patients. It supplemented the deficiencies of SOFA score (focusing on organ function) and laboratory indicators (focusing on a single pathway) from the broader dimension of physiological reserve and strike, making the evaluation level of the model more three-dimensional and complete. Lactate was included as a rapid, objective and strong prognostic biomarker of physiological disorders (Gomez and Kellum, 2015). It supplemented the assessment of tissue perfusion and cellular shock dimensions by SOFA score (organ function) and APACHE II score (comprehensive physiology), and was a key link in the multi-dimensionality of the model. The introduction of IP-10 was an innovative highlight of the model. It successfully incorporated the core pathological dimension of immune dysregulation into the prediction system, and was the key for the model to achieve excellent discrimination. In this study, the unique immune predictive value of IP-10 was systematically combined with commonly used clinical evaluation tools such as PCT, lactate, SOFA score, and APACHE II score to construct a comprehensive new nomogram model with both theoretical basis and practical potential.

The nomogram model had significant advantages. Doctors could quickly predict the risk of death for patients by integrating common clinical indicators and evaluation tools, which played a crucial role in making precise decisions regarding ICU admission, consultation, and treatment upgrades (Jin et al., 2025). Decision curve analysis confirmed that within a reasonable risk threshold range, using this model for decision-making may bring benefits to clinical treatment(Hu et al., 2025).

This model had several important clinical applications in the emergency department. First, it could identify high-risk patients who required close monitoring or active intervention through early risk stratification. Second, provided support for the admission decisions of ICU patients through precise resource allocation. Third, communicated with patients and their families to provide visual assistance for setting up practical treatment plans.

Our research also had certain limitations. First, single-center retrospective study might introduce selection bias, as the enrollment criteria and clinical management protocols employed at our institution might not fully reflect practices at other centers. Meanwhile, the demographic homogeneity of our study population—confined to a single geographic region and ethnic group—might limit the extrapolation of our findings to more diverse settings. Accordingly, a prospective multi-center validation cohort study will be required to confirm and extend our results. Second, ELISA turnaround time was not suitable for emergency settings. It should be underscored that the IP-10 assay used in the present study, being an ELISA-based method, had an inherent turnaround time of 4–6 hours and depended on standard laboratory equipment and personnel. These practical constraints precluded its use for immediate clinical decision-making at the bedside. Thus, to facilitate future clinical implementation, the development of rapid and user-friendly diagnostic platforms for IP-10 detection will be indispensable. Third, no external validation (only internal bootstrap). Our model’s predictive performance was assessed solely through internal bootstrap validation. While this approach provided some indication of internal robustness, it might not accurately reflect performance in novel patient populations. Therefore, external validation using an independent cohort from a distinct geographic region is imperative before the model can be considered for clinical implementation. We are actively planning a prospective multi-center validation cohort study to address this critical need.

As the new Surviving Sepsis Campaign guidelines have recently been released, the era of sepsis 4.0 has arrived. For acutely ill patients in hospital, NEWS (National Early Warning Score), NEW2 (National Early Warning Score 2), MEWS (Modified Early Warning Score) or SIRS (systemic inflammatory response syndrome) is strong recommended over qSOFA as a single tool to screen for sepsis (Prescott et al., 2026).

Therefore, in future research within the era of sepsis 4.0, NEWS, NEW2, MEWS, or SIRS for screening and managing early sepsis should be used. More importantly, external validation through collaboration with hospitals across different regions, along with prospective multi-center clinical validation, will be essential to ensure the robustness and generalizability of our findings.

## 5 Conclusions

For the first time, we established and validated a nomogram model for 28-day mortality based on IP-10 and clinical parameters in septic patients in the ED. Our research model provided significant value for the identification, calibration, and clinical utility of sepsis, providing support and assistance for emergency physicians in early risk stratification, resource allocation, and clinical decision-making.

## Data Availability

The original contributions presented in the study are included in the article/**Supplementary Material**. Further inquiries can be directed to the corresponding authors.
