# Supplementary material for: Development and validation of a prognostic nomogram model for 28-day mortality based on IP-10 and clinical parameters in septic patients in the emergency department
Source: Front Cell Infect Microbiol. 2026 Sep 7;16:1838120. doi: 10.3389/fcimb.2026.1838120 (PMC13590294; doi:10.3389/fcimb.2026.1838120)
Supplement: Supplementary file 1 [file DataSheet1.docx]

**Supplementary Material 1**

1. **Reading guideline of the nomogram model**

Each variable is assigned a point value based on its relative contribution to the prediction of mortality risk, allowing for a quantitative assessment of individual patient risk. From top to bottom, there are generally four types of axes. First，various variable axes (indicators). Each line represents a variable, and the scale indicates the value. Second, points axis (individual score). Shared by all variables, convert variable values into components. Third, total points axis (total score). The total score after adding up the scores of each variable. Fourth, outcome probability axis (bottom). Convert the total score into occurrence probability/risk. The common use of nomograms is divided into three steps. In the first step, each variable corresponds to a corresponding scale and the score is checked. The second step is to calculate the total score and add up the scores of all variables. The third step is that the total score corresponds to the probability and is connected from the total score axis to the outcome axis.

2. **Model application cases**

**Case Example 1: Low-Risk Patient**

**Patient A:** IP-10 40.1 (pg/ml), PCT 0.5 (ng/ml), Lac 1.5 (mmol/L), SOFA score 2, APACHE II score 6.

**Step 1**

| **Variable** | **Patient value** | **Points** |
| --- | --- | --- |
| IP-10 (pg/ml) | 40.1 | 6 |
| PCT (ng/ml) | 0.5 | 2 |
| Lac (mmol/L) | 1.5 | 4 |
| SOFA | 2 | 5 |
| APACHE II | 6 | 8 |

**Step 2**: Sum the points=6+2+4+5+8=25 Total Points

**Step3**: On the “total points” scale(bottom), find 25. Draw straight down to the “28-day Mortality Probability” axis.

**Results**: < 5% predicted 28-day Mortality

**Explanation**: Patient A has a relatively low risk, with a less than 5% chance of death within 28 days.

**Case Example 2: High-Risk Patient**

**Patient B:** IP-10 201.7 (pg/ml), PCT 4.5 (ng/ml), Lac 6.5 (mmol/L), SOFA score 8, APACHE II score 17.

**Step 1**

| **Variable** | **Patient value** | **Points** |
| --- | --- | --- |
| IP-10 (pg/ml) | 201.7 | 23 |
| PCT (ng/ml) | 4.5 | 11 |
| Lac (mmol/L) | 6.5 | 16 |
| SOFA | 8 | 22 |
| APACHE II | 17 | 25 |

**Step 2**: Sum the points=23+11+16+22+25=97 Total Points

**Step3**: On the “total points” scale(bottom), find 97. Draw straight down to the “28-day Mortality Probability” axis.

**Results**: > 99% predicted 28-day Mortality

**Explanation**: Patient B has a very high risk, with a more than 99% chance of death within 28 days.
